# Intra-tumoral heterogeneity and immune responses predicts prognosis of gastric cancer

**DOI:** 10.18632/aging.202238

**Published:** 2020-11-26

**Authors:** Wanjing Feng, Yue Wang, Siyuan Chen, Xiaodong Zhu

**Affiliations:** 1 Department of Medical Oncology, Fudan University Shanghai Cancer Center, Shanghai 200032, People’s Republic of China; 2 Department of Oncology, Shanghai Medical College, Fudan University, Shanghai 200032, People’s Republic of China; 3 Department of Hematology, Zhongshan Hospital, Fudan University, Shanghai 200032, People’s Republic of China

**Keywords:** intra-tumor heterogeneity, mutant-allele tumor heterogeneity, immune response

## Abstract

Chemotherapy resistance eventually develops in patients with gastric cancer (GC). Intra-tumoral heterogeneity (ITH) refers to the intercellular genetic variations and phenotypic diversity that affect responses to drug therapy. We measured ITH using mutant-allele tumor heterogeneity (MATH) derived from whole-exome sequencing data of patients with GC in The Cancer Genome Atlas (TCGA) database. The study included 385 patients from the TCGA database with available data regarding gastrectomy, survival, and whole-exome sequencing. Further analysis was performed in 171 GC patients with available data regarding adjuvant chemotherapy. Multiple factor analysis showed that MATH was an independent predictor of OS (hazard ratio [HR], 1.432; 95% confidence interval [CI], 1.073–1.913; *P* = 0.015) in patients with GC. Moreover, MATH was also an independent predictor of OS among the 171 GC patients who received adjuvant chemotherapy (HR, 2.016; 95% CI, 1.236–3.289; *P* = 0.005). Pathway enrichment and immune cell analyses revealed significantly higher infiltration by 20 types of immune cells in the low/intermediate group, compared to the group with high MATH scores. In conclusion, low/intermediate MATH scores predicted longer OS, when compared to those with high MATH scores. The immune response was obviously upregulated in patients with GC and low/intermediate MATH scores.

## INTRODUCTION

Gastric cancer (GC) is the fourth most common malignant neoplasm and the second leading cause of cancer-related death worldwide [1]. Although surgery is considered the only curative method for GC, perioperative chemotherapy is recommended because it improves the 5-year survival rates [2]. However, chemotherapy resistance eventually develops in such patients, and the lack of a response to drugs facilitates disease progression.

Many genes are involved in carcinogenesis, tumor progression, and metastasis. However, none of them can independently predict an individual prognosis. The heterogeneity of tumors results from the cumulative effects of many related genes [3]. Intratumoral heterogeneity (ITH), which is defined as different types of malignant cells within an individual tumor that produce subgroups of tumor cells that are resistant to therapy, plays an important role in tumor development, metastasis, and treatment resistance [4, 5]. Intratumoral heterogeneity includes intercellular genetic variation and phenotypic diversity that affect gene expression, cell proliferation, metastasis, prognosis, and responses to drug therapy [3]. Therefore, analyzing ITH might provide significant information for clinical treatment and prognosis.

Historically, methods of evaluating intra-tumor heterogeneity have been laborious and difficult to translate into clinical practice. Mutant-allele tumor heterogeneity (MATH), which measures ITH derived from whole-exome sequencing data, has recently been confirmed as a reliable, quantitative, and relatively simple way to evaluate ITH. Patients with breast cancer [6, 7], colorectal [8] and neck squamous cell carcinoma [9] who have high levels of ITH assessed by MATH, have a poor prognosis and a poor tumor response to therapy.

However, the prognostic value of ITH and genomic profiles of gastric cancer have not been assessed (GC). We aimed to determine the prognostic role of intra-tumor genetic heterogeneity in GC, using published next-generation sequencing (NGS) data about GC patients from The Cancer Genome Atlas (TCGA) database. Somatic mutations were also analyzed.

## RESULTS

### Patient characteristics and MATH scores

**Figure 1:**
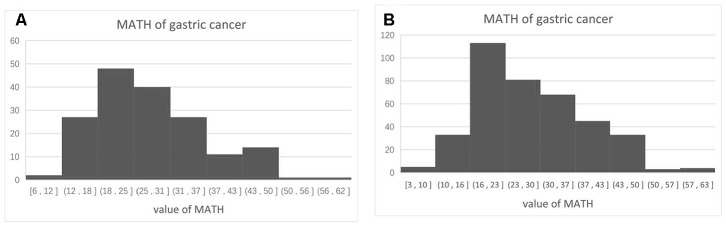
Distribution of MATH scores among 385 patients with surgically treated gastric cancer (**A**) and 171 patients treated by surgery and adjuvant chemotherapy (**B**).

The median age of the 385 patients was 67 (30–90) years. The median value of MATH was 26.2 (2.9–63.4). The clinical characteristics of the patients are described in Table 1. We divided them into low, intermediate, and high MATH groups, based on median MATH scores of 18.3 (n = 128), 24.8 (n = 128), and 38.4 (n = 128). The distribution of the MATH values is shown in Figure 1A. Univariate analysis showed that age, stage, radiation therapy, and MATH scores were significantly related to the OS (Table 1).

**Table 1:** Univariate analysis of associations between clinicopathological factors and survival in 385 patients with gastric cancer.

**Characteristics**	**Number of patients**	**P value (OS)**
Sex		0.617
Male	252(65.5%)	
Female	133(34.5%)	
Age (year)		0.016
≥60	265(68.8%)	
< 60	120(31.2%)	
Stage		0.000
I	49(12.7%)	
II	122(31.7%)	
III	162(42.1%)	
IV	36(9.4%)	
NA	16(4.2%)	
Histological Grade		0.324
Gx	9(2.3%)	
G1	10(2.6%)	
G2	144(37.4%)	
G3	222(57.7%)	
Radiation therapy		0.002
Yes	44(11.4%)	
No	156(40.5%)	
NA	185(48.1%)	
Histological type		0.239
Diffuse type	53(13.8%)	
Intestinal type	184(47.8%)	
Signet ring type	12(3.1%)	
NA	136(35.3%)	
MATH score		0.040
low	128(33.2%)	
intermediate	128(33.2%)	
high	129(33.6%)	

The median age of the 171 enrolled patients with adjuvant chemotherapy was 64 (30–90) years. The median MATH value was 25.7 (5.6–60.8). The clinical characteristics of these patients are described in Table 2. We assigned the patients as low, intermediate, and high MATH groups, based on median MATH values of 18.0, 25.8, and 37.0, respectively (n = 57 patients per group). The distribution of the MATH scores are shown in Figure 1B. Univariate analysis showed that MATH scores were significantly related to the OS (Table 2).

**Table 2:** Univariate analysis of associations between clinicopathological factors and survival in 171 patients with gastric cancer who received adjuvant chemotherapy.

**Characteristics**	**Number of patients**	**P value (OS)**
Sex		0.704
Male	117(68.4%)	
Female	54(31.6%)	
Age (year)		0.070
≥60	105(61.4%)	
< 60	66(38.6%)	
Stage	0.063
I	9(5.3%)	
II	56(32.7%)	
III	79(46.2%)	
IV	17(9.9%)	
NA	10(5.8%)	
Histological Grade	0.783
Gx	4(2.3%)	
G1	1(0.6%)	
G2	66(38.6%)	
G3	100(58.5%)	
Radiation therapy		0.058
Yes	40(23.4%)	
No	70(40.9%)	
NA	61(35.7%)	
Histological type		0.627
Diffuse type	32(18.7%)	
Intestinal type	83(48.6%)	
Signet ring type	4(2.3%)	
NA	52(30.4%)	
MATH score		0.047
low	33(33.3%)	
intermediate	33(33.3%)	
high	33(33.3%)	

### MATH and clinical outcomes

**Figure 2:**
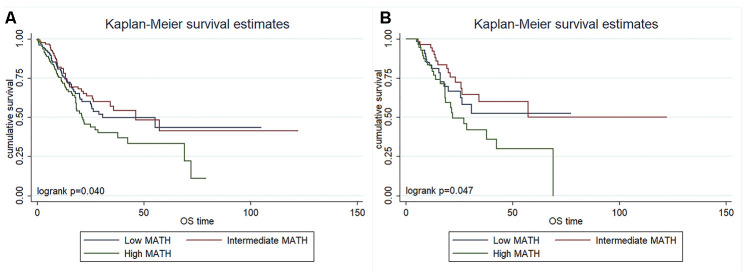
Kaplan-Meier survival overall survival curves in 385 patients with surgically treated gastric cancer (**A**) and 171 patients treated with surgery and adjuvant chemotherapy (**B**) according to MATH scores.

We determined the prognostic value of MATH scores by estimating survival using Kaplan-Meier curves and differences among three groups were analyzed using log-rank tests. Among the 385 patients, the OS was significantly shorter for those with high than low/intermediate MATH scores (*P*= 0.040; Figure 2A). The results of the multivariate Cox proportional hazards model indicated that the MATH score was an independent prognostic factor for OS (hazard ratio [HR], 1.433; 95% confidence interval [CI], 1.073-1.914; *P* = 0.015; Table 3), after adjusting for the clinicopathological characteristics of age, sex, pathological stage, radiation therapy, histological type, and grade.

**Table 3:** Multivariate Cox regression analysis of prognostic factors for overall survival in 385 patients with gastric cancer (MATH as categorized variate).

**Characteristics**	**Overall survival**	
	**Hazard ratio (95% CI)**	**P value**
age	2.066(1.163-3.670)	0.013
sex	1.119(0.695-1.801)	0.643
MATH	1.433(1.073-1.914)	0.015
pathological stage	1.736(1.288-2.341)	0.000
pathological grade	1.376(0.904-2.093)	0.136
radiation	0.418(0.227-0.767)	0.005
histological type	1.047(0.792-1.384)	0.745

Furthermore, OS was assessed in the 171 patients who had received adjuvant chemotherapy. The results also showed a significantly shorter OS among those with high scores, when compared to those with low/intermediate MATH scores (p = 0.047; Figure 2B). Results of the multivariate Cox proportional hazards model also indicated that MATH was an independent prognostic factor for OS (HR, 2.308; 95% CI, 1.300-4.097; *P* = 0.004; Table 4), after adjusting for the clinicopathological characteristics including age, sex, pathological stage, radiation therapy histological type, and grade. Therefore, a high MATH score can be considered as an independent risk factor that predicts OS in patients with gastric cancer.

**Table 4:** Multivariate Cox regression analysis of prognostic factors for overall survival in 171 patients with gastric cancer who received adjuvant chemotherapy (MATH as categorized variate).

**Characteristics**	**Overall survival**	
	**Hazard ratio (95% CI)**	**P value**
age	1.701(0.702-4.117)	0.239
sex	1.466(0.624-3.438)	0.379
MATH	2.308(1.300-4.097)	0.004
pathological stage	1.152(0.608-2.184)	0.663
pathological grade	1.193(0.560-2.538)	0.647
radiation	0.618(0.273-1.397)	0.248
histological type	1.582(0.641-3.899)	0.319

### MATH score and pathway enrichment

**Figure 3:**
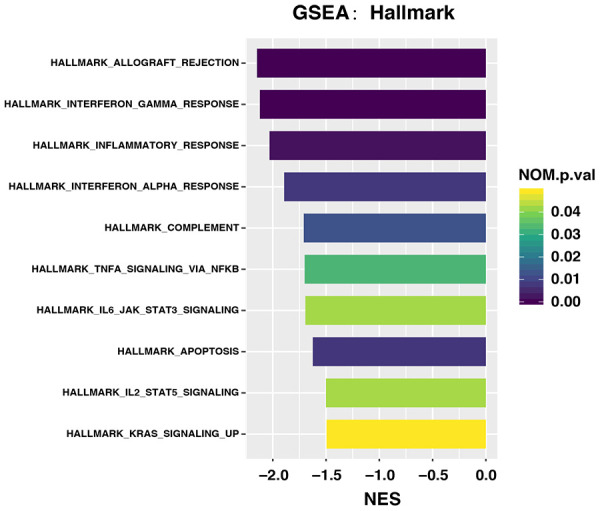
**Upregulated pathways in groups with different MATH scores among 171 patients with gastric cancer treated by surgery and adjuvant chemotherapy.**

We analyzed the differences in gene expression between patients with high and low/intermediate MATH scores using GSEA. We found 11 significantly upregulated pathways in the group with low/intermediate MATH scores, and none in the group with high MATH scores (Figure 3). The significantly upregulated immune pathways in the group with low/intermediate MATH scores included interferon gamma (IFN-γ) response, allograft rejection, inflammatory response, interferon alpha (IFN-α) response, and TNFA signaling via NFKB and complement pathways.

### Immune cell infiltration and MATH scores

We analyzed the abundance of 28 immune cell populations in the 171 patients treated with adjuvant chemotherapy. Multivariate Cox proportional hazards model analysis showed that effector memory CD8 T cells, neutrophils, immature B cells, and T follicular helper cell infiltration were significantly associated with OS (Supplementary Table 1). Wilcoxon (Mann-Whitney) tests identified 20 significantly different types of infiltrative immune cells between the high and low/intermediate MATH groups (Table 5) that comprised activated CD4 T, activated CD8 T, central memory CD4 T, central memory CD8 T, effector memory CD4 T, effector memory CD8 T, Type 1 T helper, activated dendritic, natural killer, regulatory T, type 2 T helper, gamma delta, activated B, immature B, T follicular helper, mast and myeloid-derived suppressor cells, as well as macrophages, eosinophils, and monocytes. Furthermore, infiltration by these immune cells was significantly more abundant in the group with low/intermediate, than in the group with high MATH scores.

**Table 5:** Difference of immune cell infiltration between high MATH and low/intermediate MATH group.

**Immune cell**	**z**	**P value**
Activated CD4 T cell	3.124	0.0018
Activated CD8 T cell	4.425	0.0000
Central memory CD4 T cell	2.833	0.0046
Central memory CD8 T cell	3.881	0.0001
Effector memory CD4 T cell	4.328	0.0000
Effector memory CD8 T cell	4.995	0.0000
Type1 T helper cell	5.399	0.0000
Type17 T helper cell	0.435	0.6634
Activated dendritic cell	2.866	0.0042
cd56bright natural killer cell	1.424	0.1546
Natural killer cell	3.856	0.0001
Natural killer T cell	2.774	0.0055
Regulatory T cell	4.054	0.0001
Type2 T helper cell	3.558	0.0004
cd56dim natural killer cell	-0.513	0.6078
Immature dendritic cell	1.417	0.1566
Macrophage	2.996	0.0027
MDSC	3.847	0.0001
Neutrophil	0.562	0.5743
Plasmacytoid dendritic cell	0.839	0.4014
Activated B cell	3.995	0.0001
Gamma delta T cell	2.961	0.0031
Immature B cell	4.494	0.0000
Memory B cell	0.607	0.5439
T follicular helper cell	2.382	0.0172
Eosinophil	4.057	0.0000
Mast cell	3.509	0.0004
Monocyte	2.911	0.0036

### Somatic mutations and copy number alterations

**Figure 4:**
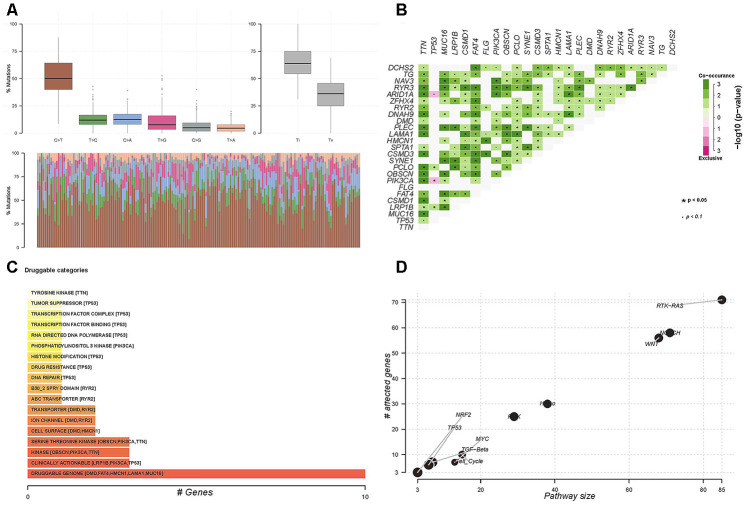
**Somatic mutations in 171 patients treated by surgery and adjuvant chemotherapy.** (**A**) Co-occurring and exclusive genes in mutation profiles. (**B**) Potential druggable gene categories and top five involved genes. (**C**) Enrichment of known oncogenic signaling pathways in patients with gastric cancer (**D**).

A missense mutation was the most prevalent type of somatic mutation among the 171 patients, and the median number of variants was 63 (Supplementary Figure 1). The mutation rates of TTN, TP53, MUC16, LRP1B, FAT4, CSMD1, SYNE1, CBSCN, FLG, and PIK3CA were the highest in the patients (Supplementary Figure 1). A model of base mutations in each patient is described in Figure 4A.

We also compared the mutation load between patients with gastric cancer and 30 other cohorts with cancer in the TCGA database. Mutations were moderate in gastric cancer patients compared with patients with other tumors (Supplementary Figure 2). We also identified potentially altered gene sets involving >2 genes that co-occur or have an exclusive mutation profile using pairwise Fisher exact tests (P<0.05) (Figure 4B).

In addition, we analyzed drug-gene interactions. The potential druggable gene categories are shown in Figure 4C and the top five genes involved in them included *FAT4*, *DMD*, *HMCN1*, *LAMA1*, and *MUC16*. We analyzed the enrichment of known oncogenic signaling pathways. Results showed that TP53, TGF-beta, MYC, NRF2, WNT, and RTK-RAS signaling pathways were enriched in the 171 patients who had received adjuvant therapy for gastric cancer (Figure 4D).

## DISCUSSION

We measured ITH using MATH. We found that OS was significantly shorter among patients with high, when compared to those with low ITH, and that MATH is an independent predictor of OS among patients with gastric cancer.

Over 70% of the patients studied herein had stage II/III gastric cancer. Adjuvant chemotherapy can improve the survival of patients with stage II/III operative gastric cancer. In Classic research which enrolled patients with stage II/III gastric cancer who underwent D2 gastrectomy, the estimated 5-year survival was significantly better in a group given adjuvant chemotherapy with oxaliplatin plus capecitabine for 6 months than in the group treated by surgery alone (78% vs. 69%) [10]. ACTS-GC study similarly showed higher 5-year OS rates in a group given adjuvant chemotherapy (S-1 for one year) compared with a group treated only by surgery (71.1% vs. 53.1%). Although the estimated 5-year survival rate increased after surgery plus adjuvant chemotherapy compared with surgery alone, chemotherapy did not confer a survival benefit on quite a few patients [11].

According to our findings, ITH plays an important role in the clinical outcomes of patients with gastric cancer. This was probably because high ITH caused primary resistance to chemotherapy. Clinical experience has shown that a progression-free survival benefit does not always translate into an OS benefit [12]. Therapy might eliminate the dominance of sensitive clones, resulting in the acceleration of drug-resistant subclones with the release of competition and source-rich environment [13]. Therefore, we speculated that the survival of patients with high MATH scores cannot benefit from adjuvant chemotherapy. We then analyzed differences in pathway enrichment and immune cell infiltration between the groups with high and low MATH scores.

We found that immune cell infiltration strikingly differed between them. Notably, 20 types of immune cells that differed between the two groups; increased infiltration was evident in the low/intermediate MATH group, regardless of the presence of anti- or pro-tumor immune cells. Moreover, pathway enrichment analysis also showed that several immune pathways were upregulated among the low/intermediate, compared with the high MATH group. These pathways included responses to interferon (IFN)-γ, IFN-α, and inflammation, as well as TNF α signaling via NFκB, and complement. Interferon-γ and IFN-α are both associated with anti-tumor processes and immunoregulation [14–16]. Interferon-α stimulates both macrophages and NK cells [17], and it has been approved for use against follicular lymphoma and hairy-cell leukemia [18, 19]. Interferon-γ is involved in both innate and adaptive immune responses, which include anti-viral, anti-tumor, and immunoregulatory properties [14, 20–22]. Interferon-γ stimulates macrophages to induce anti-tumor mechanisms and the upregulation of antigen presentation. An *in vitro* study found that IFN-γ is associated with the inhibition of cell proliferation and cell death [19]. Although IFN- γ has not yet been approved for medical treatment, it has improved the survival of bladder carcinoma in clinical trial [18]. The complement system is an important part of innate immunity. Complement activation is considered an antitumor process for two reasons; the complement system is an important part of immune surveillance, and complement-dependent toxicity is considered the main mechanism of antitumor monoclonal antibodies, such as rituximab in diffuse large B cell lymphoma (DLBCL) [23, 24]. Inflammation plays an important role in tumorigenesis [25]. The INF family, TNF-α and the complement system are all involved in the inflammatory response [26].

Our pathway enrichment and immune cell infiltration findings revealed that the immune response was significantly upregulated in the low/intermediate MATH group. That the immune response is upregulated can be inferred, as clinical outcomes were better in the group with low/intermediate MATH scores. However, whether adjuvant chemotherapy activates the immune response requires further validation.

This study has some limitations. Our study did not include a validation cohort. We queried all public databases; however, another cohort of patients with gastric cancer with whole-exome sequencing data, Mutation Annotation Format (maf) files, and documented follow-up durations was not found. Therefore, the relationship between ITH and the immune response requires further investigation.

In conclusion, the MATH score, which represents ITH, is an independent prognostic factor for patients with gastric cancer treated by gastrectomy and adjuvant chemotherapy. The OS is significantly longer among patients with low, than those with high ITH.

## MATERIALS AND METHODS

### Patient enrolment

Whole-exome sequencing (~1% of the genome, at 150-fold mean sequence coverage), clinical characteristics, and follow-up duration were obtained from TCGA database using the Cancer Genomics Browser of the University of California Santa Cruz (https://xena.ucsc.edu/welcome-to-ucsc-xena/). Patients, for whom whole-exome sequencing data, Mutation Annotation Format (maf) files, and follow-up records were unavailable, were excluded. In total, we analyzed the survival of 385 patients with GC. We also analyzed 171 patients with GC who had complete chemotherapy information were included in further analysis. The main outcome measurement was overall survival (OS) defined as the interval between the date of diagnosis and the date of death.

### Mutant allele tumor heterogeneity

The mutant allele tumor heterogeneity (MATH) algorithm was used to measure ITH. The calculation method of MATH for TCGA was identified at the Broad Institute of MIT and at Harvard. We obtained difference values of the MAF from the median difference value. The median absolute deviation (MAD) in R was then calculated as the value scaled by a factor (1.4826) to render the expected MAD of a sample from a normal distribution equal to the standard deviation. The MATH score was calculated as MATH = 100 9 MAD/median. We also used the maftools package in R to calculate MATH, which includes a clustering algorithm to improve the accuracy of genomic profiles.

### Pathway enrichment analysis

We identified upregulated pathways among MATH groups using gene set enrichment analysis (GSEA) of adjusted RNA-Seq data [27]. Significance was identified according to the following standards: a nominal value of p < 0.05, an NES value of ≥ 1 and an FDR q value of < 0.25. We downloaded gene sets from the MSigDB database [27].

### Estimation of immune cell infiltration

We estimated the abundance of 28 types of immune cells from RNA-seq data of each sample using gene set variation analysis (GSVA). Immune cell populations were identified by gene sets overexpressed in each type of immune cell [28, 29]. The GSVA scores of immune cells between patients with high and low/intermediate MATH scores were compared using Wilcoxon (Mann-Whitney) tests.

### Statistical analysis

Values with two-sided P < 0.05 were considered significant. Patient baseline characteristics were compared according to MATH scores using one-way analyses of variance (ANOVA) OS was compared between groups using log-rank tests. Prognostic predictors were assessed by multivariate analysis using Cox proportional hazards models. All data were statistically analyzed using R version 3.5.1 (http://cran.r-project.org) and Stata Statistical software, version 12.0 (StataCorp Llc., College Station, TX, USA).

### Ethical approval

This article does not contain any studies with human participants or animals.

## Supplementary Material

Supplementary Figures

Supplementary Table 1
