# Supplementary material for: Intra-tumoral heterogeneity and immune responses predicts prognosis of gastric cancer
Source: Aging (Albany NY). 2020 Nov 26;12(23):24333–44. doi: 10.18632/aging.202238 (PMC7762511; doi:10.18632/aging.202238)
Supplement: Supplementary Figures [file aging-12-202238-s001.pdf]

SUPPLEMENTARY FIGURES

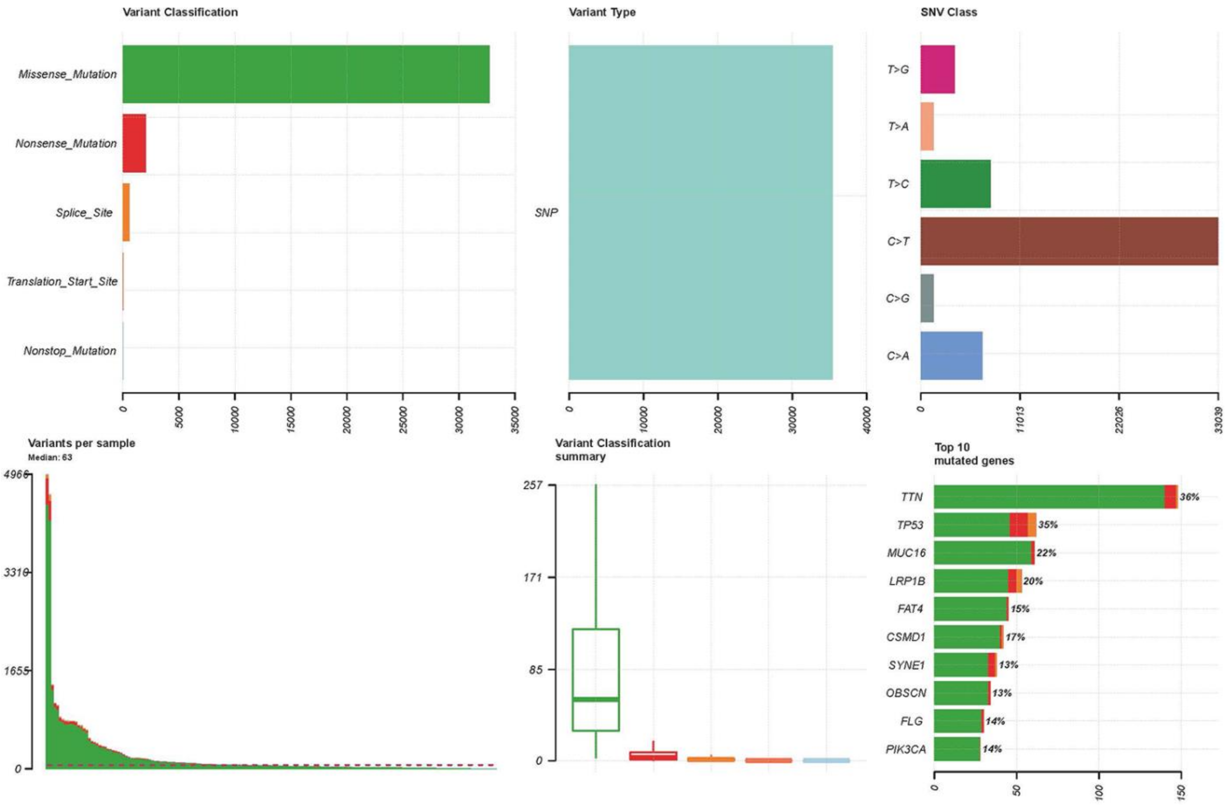

Supplementary Figure 1. Summary of somatic mutations in 171 patients with gastric cancer.

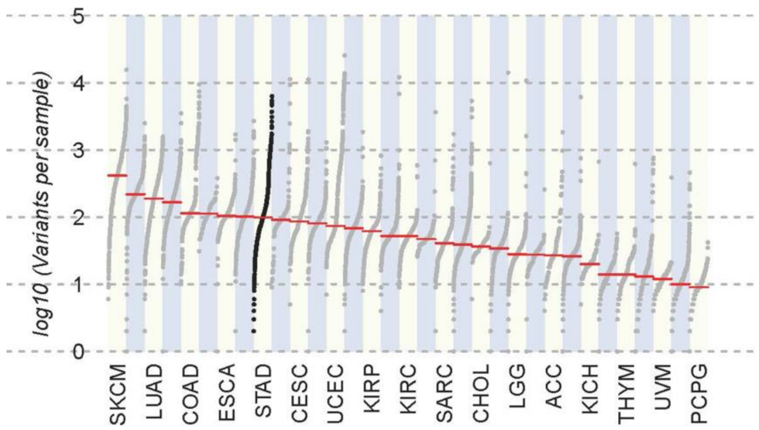

Supplementary Figure 2. Mutation load of 31 cancer cohorts in TCGA database.
