# Supplementary material for: Intra-tumoral heterogeneity and immune responses predicts prognosis of gastric cancer
Source: Aging (Albany NY). 2020 Nov 26;12(23):24333–44. doi: 10.18632/aging.202238 (PMC7762511; doi:10.18632/aging.202238)
Supplement: Supplementary Table 1 [file aging-12-202238-s002.pdf]

SUPPLEMENTARY TABLE

Supplementary Table 1. Multivariate Cox regression analysis of immune cells for overall survival in the 171 gastric cancer patients with adjuvant chemotherapy.

Characteristic	Overall Survival	
	Hazard ratio (95% CI)	P value
Activated CD4 T cell	1.794(0.130-24.628)	0.662
Activated CD8 T cell	1.200(0.031-45.277)	0.921
Central memory CD4 T cell	1.569(0.157-15.653)	0.701
Central memory CD8 T cell	0.228(0.021-2.420)	0.220
Effector memeory CD4 T cell	0.312(0.031-3.061)	0.317
Effector memeory CD8 T cell	153.365(2.689-8745.239)	0.015
Type 1 T helper cell	0.056(0.000-4.660)	0.202
Type 17 T helper cell	0.724(0.130-4.008)	0.712
Activated dendritic cell	0.432(0.031-5.992)	0.532
CD56bright natural killer cell	0.344(0.059-2.000)	0.235
Natural killer cell	0.432(0.030-6.133)	0.535
Natural killer T cell	1.301(0.079-21.361)	0.854
Regulatory T cell	0.328(0.016-6.590)	0.467
Type 2 T helper cell	1.344(0.185-9.748)	0.770
CD56dim natural killer cell	0.983(0.136-7.078)	0.987
Immature dendritic cell	0.227(0.042-1.217)	0.084
Macrophage	0.646(0.030-13.776)	0.780
MDSC	1.141(0.014-89.158)	0.953
Neutrophil	9.599(1.244-74.022)	0.030
Plasmacytoid dendritic cell	1.916(0.188-19.473)	0.583
Activated B cell	11.444(0.159-820.920)	0.264
Gamma delta T cell	0.915(0.090-9.204)	0.940
Immature B cell	0.002(0.000-0.974)	0.049
Memory B cell	1.453(0.291-7.251)	0.648
T follicular helper cell	47.155(3.699-600.992)	0.003
Eosinophil	0.284(0.046-1.742)	0.174
Mast cell	7.171(0.583-88.156)	0.124
Monocyte	1.153(0.096-13.768)	0.910
